# Are products with an orphan designation for oncology indications different from products for other rare indications? A retrospective analysis of European orphan designations granted between 2002-2012

**DOI:** 10.1186/s13023-017-0578-4

**Published:** 2017-02-16

**Authors:** Kim Pauwels, Isabelle Huys, Minne Casteels, Kristina Larsson, Caroline Voltz, Karri Penttila, Thomas Morel, Steven Simoens

**Affiliations:** 1 Department of Pharmaceutical and Pharmacological Sciences, KU Leuven, 3000 Leuven, Belgium; 2 European Medicines Agency, London, UK; 3 Finnish Medicines Agency, Helsinki, Finland; 4 Savonlinna Central Hospital, Savonlinna, Finland

**Keywords:** Orphan designation, Orphan medicinal product, Neoplastic disorders, Cancer, EMA

## Abstract

**Background:**

Orphan designated medicinal products benefit from regulatory and economic incentives for orphan drug development. Approximately 40% of orphan designations target rare neoplastic disorders, referring to rare cancers. In order to provide more insights in drugs for rare neoplastic disorders that are under development and to better understand the role of orphan designation in the development of oncology drugs, this study investigates the characteristics of the product, the indication and the applicants as well as the stage of development of products with an orphan designation for rare neoplastic disorders and compares them with products with an orphan designation for other rare indications. Therefore, orphan designation application files and annual reports submitted by the applicant were reviewed at the premises of the European Medicines Agency.

**Results:**

At the time of application, 41.6% of products with orphan designation for rare neoplastic disorders were in pre-clinical phase; this was 65.1% for other rare conditions (*p* < 0.05). Thirty percent of orphan designations for rare neoplastic disorders had reached phase 1; compared to 19.3% of orphan designations targeting other rare conditions (*p* < 0.05). The same trend was observed for the stage of development at the time of the latest annual report. Significant benefit was more often considered for orphan designations for rare neoplastic disorders compared to orphan designations for other rare conditions.

**Conclusion:**

Orphan designations for rare neoplastic disorders involve products that are in a more advanced stages of development compared to orphan designations for other (non-oncology) rare conditions.

## Background

Rare cancers affect around 4.3 million persons in the European Union (EU) and more than 500,000 new cases are diagnosed every year [1]. Despite the small number of patients per indication, rare cancers now represent a significant number of the total burden of cancer, with 22% of all cancer cases diagnosed in the EU each year considered as ‘rare’ [2].

In 2000, the European Commission (EC) introduced the Regulation on Orphan Medicinal Products (OMP) to provide regulatory and economic incentives for orphan drug development [3]. OMP designations can be assigned to medicinal products prior to marketing authorization, on the condition that the product is intended for diagnosis, prevention or treatment of a life-threatening or chronically debilitating condition that affects less than 5 in 10,000 persons in EU or when marketing is unlikely due to insufficient return on investment. Further, should there be no satisfactory method of diagnosis, prevention or treatment of the condition that has been authorized in Europe, or if such methods exist, that the medicinal product requesting OMP designation offers significant benefits compared to existing treatments [3]. Many incentives have been introduced with the OMP legislation [3]. Medicinal products with OMP designation have access to a reduced fee for a specific scientific advice procedure, called protocol assistance. Whereby scientific advice provides a sponsor with guidance on regulatory requirements for the demonstration of quality, safety and efficacy of the drug, protocol assistance can additionally answer questions with regard to the significant benefit criterion for OMP designation. Protocol assistance is free of charge for registered Small and Medium sized Enterprises (SMEs) [3]. Further, medicinal products with OMP designation benefit from a reduction in the regular fee for the centralized procedure for marketing authorization and are protected by a 10-year market exclusivity period from the moment that marketing authorization is granted from similar medicinal products [3]. A single medicinal product can obtain an OMP designation for multiple conditions and an OMP designation can be granted to multiple medicinal products targeting the same oncological indication. When designated as an OMP, sponsors are obliged to provide yearly reports on progress of drug development [3]. By 2015, over 1500 OMP designations were granted based on a positive opinion from the Committee for Orphan Medicinal Products (COMP), over 100 OMPs received marketing authorization in Europe.

The high burden of rare cancers shows the need for accessible and effective drugs to treat these diseases. A previous study already showed a promising OMP pipeline where rare neoplastic disorders represent an important share of the indications, however, the pipeline for OMP that intent to treat cancer was not specifically investigated in this study [4]. OMP designation seems an important track for oncology drug development since approximately 40% of all OMP designations and an equal number of authorized OMPs target rare cancers [4, 5]. This is in contrast with the proportion of medicinal products for non-orphan conditions, where oncology products only represent 13% of the total number of drugs [6]. In order to better understand the role of OMP designation in the development of oncology drugs, this study investigates the characteristics of the product, the indication and the applicants as well as the stage of development for OMP designations for rare cancer and compares them with OMP designations for other rare indications.

## Methods

The data for this study were collected in the context of an analysis on the orphan drug pipeline across all indications. Additional methodological details can be found elsewhere [4].

OMP designations fulfilling the following conditions were included in this study: OMP designation was granted between January 1st 2002 and December 31st 2012, designation was valid on June 13th 2014, no marketing authorization was granted by the EC over the study period. The Community Register of OMP for human use and Orphadata, the scientific dataset developed and managed by Orphanet, were consulted to determine the study sample. Data were collected at the premises of the European Medicine Agency (EMA) in London during November 2014.

The following variables were used for this study: year of application, designation year, designated orphan indication, Orphanet^©^ linearization disease category, type of product (gene therapy, ingredient/substance, cell therapy product, human/animal tissue/organ, blood derived product) and type of production (e.g. biotechnology or synthetic/extractive chemistry), applicant categorization (academia/public body, physical persons, consulting, small pharma, medium pharma, large pharma or SME), prevalence of the indication, use of significant benefit criterion by EMA at the time of application, status of drug development at time of application and at the time of the latest annual report.

Applicants were categorized based on Amadeus database [4]. Academia/public bodies, consulting and physical persons were identified by website search. The Amadeus© (Bureau van Dijk) database of financial and business information was consulted to categorize private sponsors based on annual operating revenue. Sponsors with an annual operating revenue above €25 billion were assigned to the group of very large pharmaceutical companies. If the annual operating revenue was between €24 billion and €6 billion, the sponsor was categorized as being “large”. Less than €5 billion revenue was allocated to “intermediate sized companies” and less than €50 million was allocated in “small or medium-sized enterprise” (SME). SME’s are defined by the EC based on turnover and staff headcount, however, in this study, staff headcount was not considered for the categorization.

The prevalence of the indication was retrieved from the application file submitted by the applicant to EMA and segmented in three categories i.e. high prevalence (>3/10,000), medium prevalence (1–3/10,000) and low prevalence (<1/10,000).

When designated as an OMP, sponsors are obliged to provide yearly reports on progress of drug development. While the status of drug development at the time of OMP designation can contribute to understanding the role of OMP designation for rare neoplastic disorders, the stage of development in the annual report allows insights in the OMP pipeline for rare neoplastic disorders, complementary to a previous study on OMP pipeline in general [4]. In case data about the state of drug development could not be retrieved from the most recent annual report, the report that was provided during the previous reporting period was used. Missing data were reported if appropriate data were not available in the most recent and previous reports. Status of drug development was classified in pre-clinical research, phase 0, phase I, phase II and phase III and compassionate use (CU) studies. Annual reports are specific to each designation. As a consequence, when one active substance is the subject of several designations, a separate report was prepared for each designation.

OMP designations were categorized in a group for rare cancers, further referred as ﻿rare neoplastic disorders and a group for other rare conditions, based on Orphanet^©^ linearization disease category. The proportion of designations that belong to a certain category of product type, applicant category, prevalence category, consideration of significant benefit criterion and stage of development reported in the application file and the latest annual report were compared between designations for rare neoplastic disorders versus designations for other rare conditions using Chi^2^ test. A 0.05 confidence level was considered. Analysis was performed in IBM Statistics SPSS 23.

## Results

**Fig. 1:**
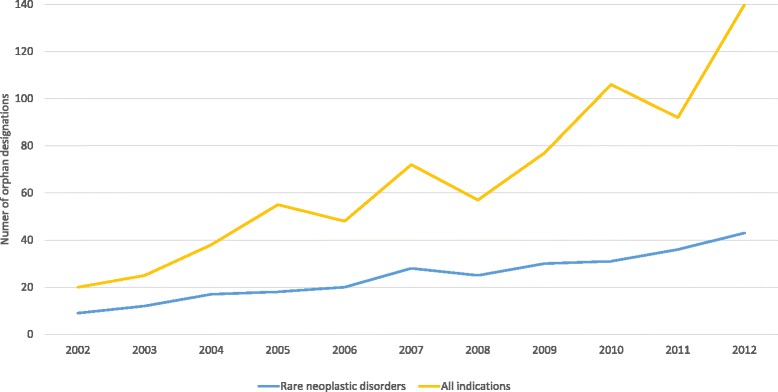
Number of orphan designations for rare neoplastic disorders in relation to the total number of designations between 2002 and 2012

Information on 730 designations was collected. Of these designations, 269 (36.8%) involved rare neoplastic disorders and 461 (63%) involved other rare conditions. The number of conditions for which positive opinion on OMP designation was granted increased over time, from 20 conditions in 2002 to 140 conditions in 2012 (Fig. 1). The proportion of ODs targeting rare neoplastic disorders over total ODs has been slightly decreasing over the study period 2002 to 2012 (Fig. 1). While in 2002, 45% of the OMP designations involved rare neoplastic disorders, only 31% of OMP designations were granted for rare neoplastic disorders in 2012.

There was no significant difference in product type between products that obtained OMP designation for rare neoplastic disorders and products that obtained a designation for other rare conditions (Table 1). More than half of the designations for rare neoplastic disorders was applied for by SME’s. Although SME’s also compose the majority of applicants for other rare conditions, their involvement was significantly lower compared to in rare neoplastic disorders (Table 1). On the other hand, designations for rare neoplastic disorders were never applied by academia or public bodies and only in singly cases applied for by physical persons. Although the number of applications from this group for other rare conditions was still small, the difference with rare neoplastic disorders was significant (Table 1).

**Table 1:** Comparison product type, applicant categorization, prevalence segmentation and consideration of significant benefit criterion for products that obtained OMP designations for rare neoplastic disorders and products that obtained OMP designation for other rare conditions

	Rare neoplastic disorders (*N* = 269)	Other rare conditions (*N* = 461)
Product type		
Synthetic/extractive agent	53.5% (144)	53.8% (248)
Biotechnology	46.5% (125)	46.2% (213)
Applicant categorization		
Academia/Public body	0% (0)^a^	4% (20)^a^
Consulting	9% (25)	11% (52)
Physical person	2% (6)^a^	6% (27)^a^
SME	56% (150)^a^	47% (216)^a^
Intermediate sized company	13% (35)	16% (75)
Large Pharma	13% (35)^a^	7% (31)^a^
Very large Pharma	7% (18)	9% (40)
Prevalence segmentation		
< 1/10,000	19% (51)	47.7%(220)
1–3/10,000	69.1% (186)^a^	41.4% (191)^a^
> 3/10,000	11.9% (32)^a^	10.8% (50)^a^
Consideration of significant benefit criterion		
Yes	75.1% (202)^a^	44.3% (204)^a^

^a^Indicates difference at 0.05 level based on Chi^2^ test

Almost 70% of rare neoplastic disorders had a prevalence of one to three persons in a population of 10,000. This was significantly different from the indication of other rare conditions as in this group, almost half of the designations involved an indication that occurred less than 1 time in 10,000 persons (Table 1).

While the significant benefit criterion was considered in 75% of OMP designations granted for rare neoplastic disorders, less than half of the designations for other rare conditions included an assessment of the significant benefit of the product (Table 1).

**Fig. 2:**
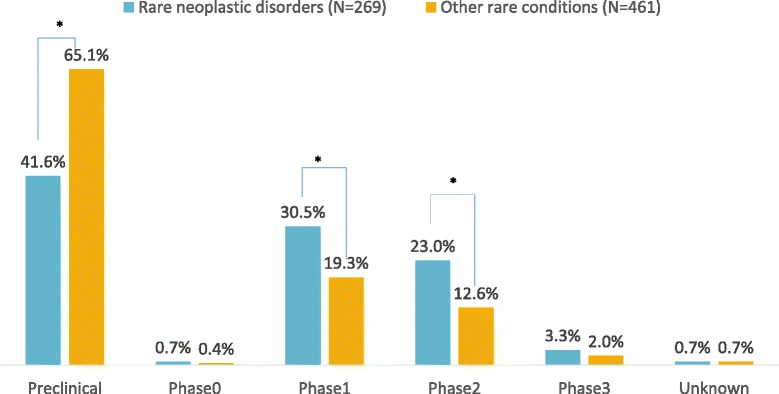
Orphan designations at time of application, described by stage of development. *Indicates significant differences at the 0.05 level

**Fig. 3:**
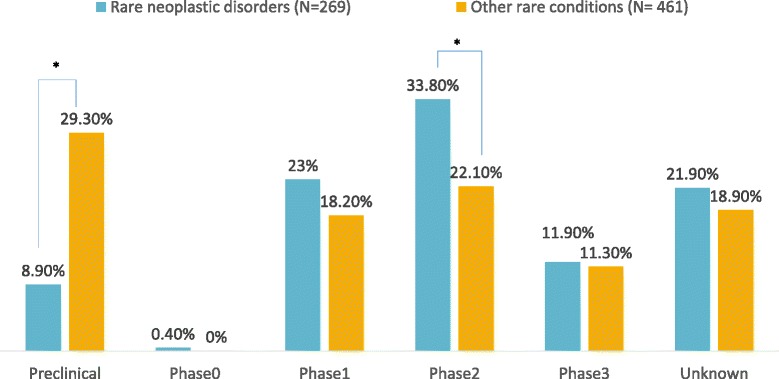
Orphan designations at time of latest annual report, described by stage of development. *Indicates significant differences at the 0.05 level

At the time of application for an OMP designation, the majority of products for rare neoplastic disorders already reached the clinical development stage. This is in contract to products for other rare indications were more than half of the products still remain in pre-clinical phases (Fig. 2). At the time of the most recent annual report, only one third of products for other rare conditions is still in pre-clinical development, however, the proportion of products for rare neoplastic disorders in pre-clinical development is less than 10% (Fig. 3).

There was no significant relationship between prevalence segmentation and stage of development.

Significant differences were observed in the stage of development of the products between different categories of applicants. OMP designations for rare neoplastic disorders were more often applied for based on phase 2 studies by SME’s, medium pharma, large pharma and very large pharma compared to physical persons. There was no significant difference across different applicant sizes for the proportion of OMP designations applied for based on non-clinical data, phase 1 or phase 3 studies (data not shown).

OMP designations for other rare conditions were more often applied for based on non-clinical data by large pharma compared to very large pharma. Medium pharma, SME, consulting, physical persons and academia more frequently used non-clinical data when applying OMP designation for other rare conditions, while there was no significant difference between very large pharma and large pharma (data not shown).

## Discussion

This study provides insights in the pipeline for OMP for rare neoplastic disorders and the role of OMP designation in the development of oncology drugs. Previous analysis showed that the majority of OMP designations across all conditions was granted a positive opinion based on preliminary clinical data with the product in patients with the condition, with only around 30% of submissions that show medical plausibility based on in vivo data only [4, 7]. Our study showed that the proportion of products in preclinical phase was much lower for rare neoplastic disorders compared to other rare indications. An analysis on the orphan drug pipeline in general estimated that between 90 and 100 products of the sample can reach marketing authorization in the future [4]. Based on the results of this study, however, it is still unsure whether the more advanced stage of development of OMP designation for rare neoplastic disorders are also associated with higher success rates for marketing authorization. The following section will discuss reasons that might explain the observed results.

Consideration of significant benefit for products that is intented to treat rare neoplastic disorders, suggest that alternative treatments exist more often for these diseases compared to other rare conditions. Over the last decade, technological evolutions in the field of microscopy, molecular biology and genomics led to improved understanding of the mechanisms behind cancer. In parallel with these technological evolutions, a competitive market with multiple therapeutic options per indication was established and today, the market is still evolving [8]. The presence of twelve centrally authorized products to treat multiple myeloma can illustrate this, and more is yet to come since multiple myeloma/plasma cell myeloma (terminology used interchangeably) is also the subject of 20 OMP designations which are still active by October 2016 [6].

Uncertainties about safety and efficacy will be less acceptable when alternative products are already available. In addition quality, safety and efficacy requirements of the drug, evidence for significant benefit is a requirement for OMP designation but also necessary to confirm at the time of market authorization. Incentives for development of OMP such as protocol assistance can support applicants at the moment they approach the stage of clinical development where they need to compete with alternative treatments for which experience is already gained after marketing authorization. Protocol assistance can also aid towards generation of appropriate data to substantiate marketing authorization later on.

Registered SME obtain free protocol assistance upon OMP designation. Our study showed a significant difference in the proportion of SME sponsoring OMP designated products, but categorization of companies is based on Amadeus database, different from criteria applied by the European Commission. Although some differences in company size were observed between sponsors of OMP designations for rare neoplastic disorders versus other conditions, there was no general trend towards larger companies in the group of rare neoplastic disorders. Our results indicate that for rare neoplastic disorders, clinical data are achievable even for smaller sized companies as phase II clinical data for rare neoplastic disorders were equally available for smaller sized companies compared to medium and large size companies. It can be hypothesizes whether the transition from pre-clinical studies towards clinical studies is easier for rare neoplastic disorders compared to other rare indications. On the one hand, this can be due to technological evolutions and abundant experience in the disease domain, allowing better understanding of neoplastic disease and mechanisms behind therapeutic agents. In some cases, clinical experience with an authorized product (which can even be the same product with a marketing authorization in another indication) using the same mechanism of action as the one applying for OD may already be available. Experiences in a broad indication can be used in limited patient populations were efficacy is increased. Conditions other than those for neoplastic disorders are often less known and less understood. On the other hand, the questions raises whether the benefit-risk ratio that is accepted within clinical trials for rare neoplastic disorders is the same for other rare neoplastic conditions.

As the reasons for the findings in this study are unknown, the authors hypothesis that competition may complicate demonstration of significant benefit based on pre-clinical data, and therefore OMP designations for rare neoplastic disorders are more likely to be applied at the stage of clinical development. This may also suggest that significant benefit for rare neoplastic disorders can go beyond mechanisms of action, tumor response, cellular toxicity and survival but involve patient relevant outcomes that can only be proven based on clinical data.

This study is subject to two limitations. First, the study data are limited to OMP designations granted between 2002 and 2012. In order to include data from the annual report, a timespan of at least one year is required between the moment that the orphan designation is granted and the moment that data are collected. Nevertheless, new orphan designations were granted in the time between the data collection and publication of these results. Second, the data of this study only include submission for orphan designation for which a positive decision was granted. Therefore, we cannot draw conclusions on factors that play a determinative role in the provision of orphan designations, neither on the success factors to prove significant benefit. An updated dataset that is extended towards unsuccessful submission for OMP designations could further contribute to understanding the role of the OMP Regulation in the development of oncology drugs and provide valuable knowledge to policymakers, payers and industry that is essential to reduce the burden of rare cancers in Europe.

## Conclusion

At time of data analysis, products with OMP designation are in a more advanced stage of development when they intent to treat rare neoplastic disorders than when they intent to treat other rare indications. The competitive character of the oncology market in combination with the requirement of significant benefit for OMP designations are a potential explanation for the observed results.

## Abbreviations

COMP: Committee of Orphan Medicinal Products
EC: European Commission
EMA: European Medicines Agency
EU: European Union
OMP: Orphan Medicinal Product
R&D: Research & Development
SME: Small and Medium sized Enterprises
